# RbCuFe(PO_4_)_2_

**DOI:** 10.1107/S1600536813019569

**Published:** 2013-07-24

**Authors:** Abdessalem Badri, Mourad Hidouri, Mongi Ben Amara

**Affiliations:** a Faculty of Science, University of Monastir, 5019 Monastir, Tunisia

## Abstract

A new iron phosphate, rubidium copper(II) iron(III) bis­(phosphate), RbCuFe(PO_4_)_2_, has been synthesized as single crystals by the flux method. This compound is isostructural with KCuFe(PO_4_)_2_ [Badri *et al.* (2011 ▶), *J. Solid State Chem.* **184**, 937–944]. Its structure is built up from Cu_2_O_8_ units of edge-sharing CuO_5_ polyhedra, inter­connected by FeO_6_ octa­hedra through common corners to form undulating chains that extend infinitely along the [011] and [01-1] directions. The linkage of such chains is ensured by the PO_4_ tetra­edra and the resulting three-dimensional framework forms quasi-elliptic tunnels parallel to the [101] direction in which the Rb^+^ cations are located.

## Related literature

For the physical properties of iron phosphates, see: Elbouaanani *et al.* (2002 ▶). For the structural chemistry of iron phosphates, see: Moore (1970 ▶); Gleitzer (1991 ▶). For rubidium iron phosphates, see: Hidouri *et al.* (2010 ▶). The title compound is isostructural with KCuFe(PO_4_)_2_ (Badri *et al.*, 2011 ▶) and K(Fe,Mg)(PO_4_)_2_ (Yatskin *et al.*, 2012 ▶). For P—O distances in monophosphate groups, see: Baur (1974 ▶). For ionic radii, see: Shannon (1976 ▶).

## Experimental

### 

#### Crystal data

RbCuFe(PO_4_)_2_
*M* *_r_* = 394.80
Monoclinic, 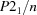

*a* = 8.054 (1) Å
*b* = 9.906 (3) Å
*c* = 9.140 (1) Å
β = 115.47 (1)°
*V* = 658.3 (2) Å^3^
*Z* = 4
Mo *K*α radiation
μ = 13.28 mm^−1^
*T* = 293 K
0.25 × 0.21 × 0.14 mm

#### Data collection

Enraf–Nonius CAD4 diffractometer
Absorption correction: part of the refinement model (Δ*F*) (*SHELXLA*; Sheldrick 2008 ▶) *T*_min_ = 0.060, *T*_max_ = 0.119
1939 measured reflections
1437 independent reflections
1236 reflections with *I* > \2(*I*)
*R*_int_ = 0.046
2 standard reflections every 120 min intensity decay: 1%

#### Refinement

*R*[*F* ^2^ > 2σ(*F* ^2^)] = 0.046
*wR*(*F* ^2^) = 0.126
*S* = 1.08
1437 reflections
54 parameters
Δρ_max_ = 3.25 e Å^−3^
Δρ_min_ = −2.25 e Å^−3^

### 

Data collection: *CAD-4 EXPRESS* (Enraf–Nonius, 1994 ▶); cell refinement: *CAD-4 EXPRESS*; data reduction: *XCAD4* (Harms & Wocadlo, 1995 ▶); program(s) used to solve structure: *SIR92* (Altomare *et al.*, 1993 ▶); program(s) used to refine structure: *SHELXL97* (Sheldrick, 2008 ▶); molecular graphics: *DIAMOND* (Brandenburg, 1999 ▶); software used to prepare material for publication: *SHELXL97*.

## Supplementary Material

Crystal structure: contains datablock(s) global, I. DOI: 10.1107/S1600536813019569/br2226sup1.cif

Structure factors: contains datablock(s) I. DOI: 10.1107/S1600536813019569/br2226Isup2.hkl

Additional supplementary materials:  crystallographic information; 3D view; checkCIF report

## Figures and Tables

**Fig. 1.**
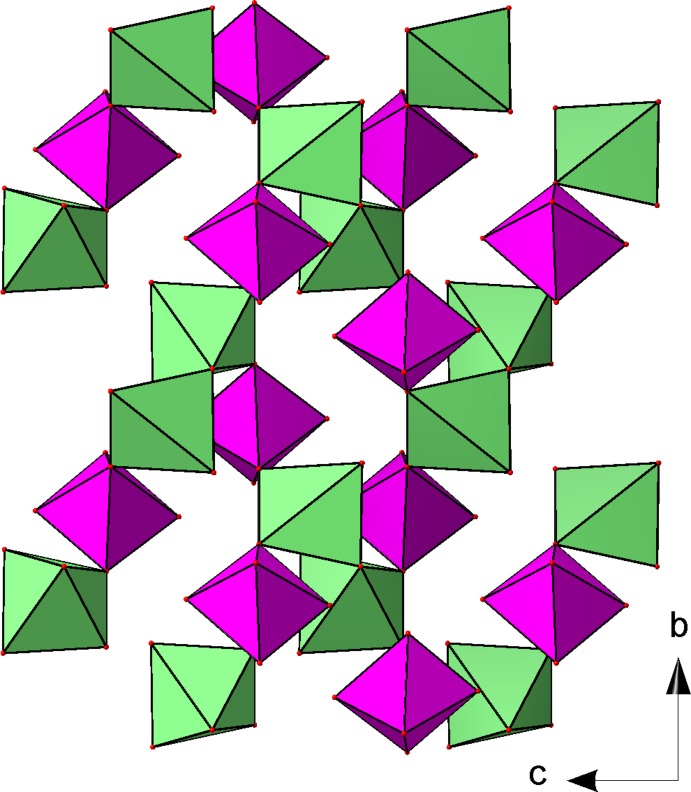
The crossing chains of the alternating Cu2O8 units and FeO6 octahedra. The FeO6 octahedra and the CuO5 trigonal bipyramids are represented by purple and green polyhedra, respectively.

**Fig. 2.**
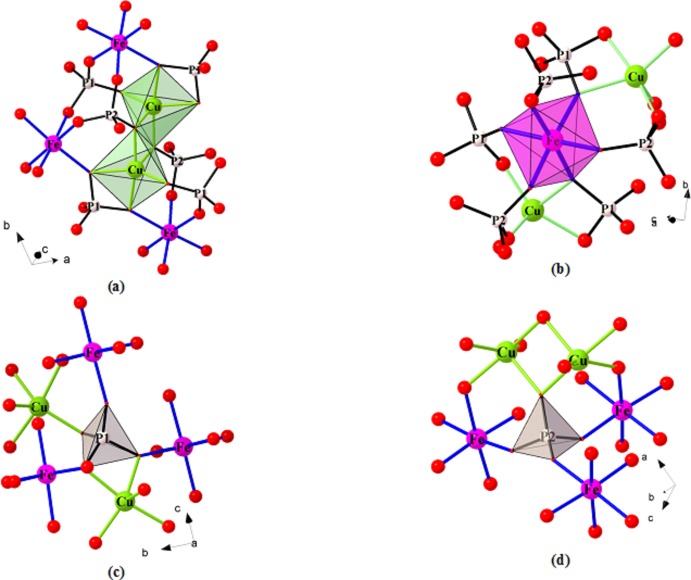
The environments of the CuO5 (a), FeO6 (b), P1O4 (c) and P2O4 (d) polyhedra.

**Fig. 3.**
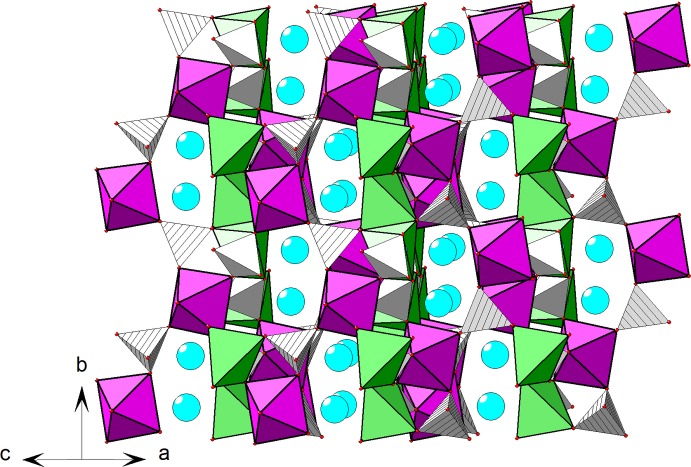
A projection along the [101] direction of the structure showing the large tunnels occupied by the Rb+ cations. FeO6, CuO5, P1O4 and P2O4 are represented by purple, green, solid grey and hatched grey polyhedra, respectively. The Rb atoms are illustrated by cyan circles.

**Fig. 4.**
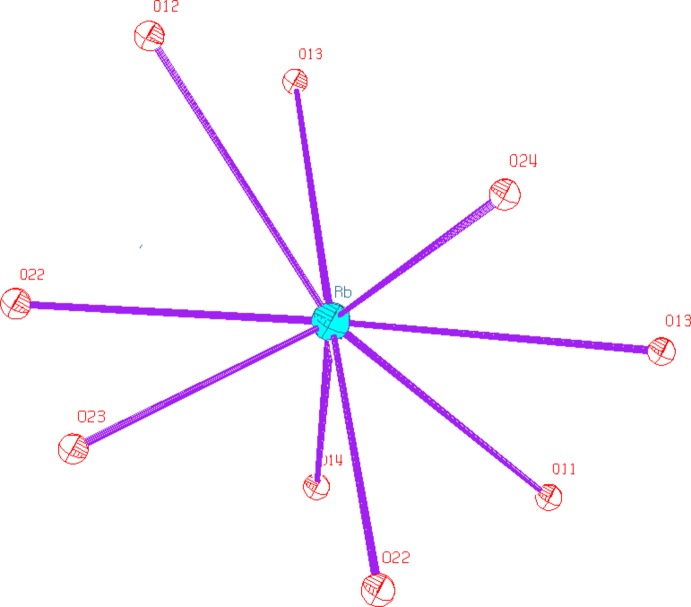
The environment of the Rb cations showing the thermal ellipsoids drawn at the 50% probability level.

## supplementary crystallographic information

### Comment

Iron phosphates are the subject of growing interest because of their attractive physical properties (Elbouaanani *et al.*, 2002) as well as for their structural diversity (Moore, 1970; Gleitzer, 1991). Among the variety of iron monophosphates synthesized and characterized over the past three decades, only one rubidium-containing compound has been reported, namely Rb_9_Fe_7_(PO_4_)_10_ (Hidouri *et al.*, 2010). In this paper, we report the structure of a new rubidium iron monophosphate RbCuFe(PO_4_)_2_ synthesized during our investigation of the Rb_3_PO_4_—Cu_3_(PO_4_)_2_-FePO_4_ quasi system.

Due to the poor quality of the crystal, anisotropic structure refinement systematically led to non-positive definite thermal parameters. For this reason, all the atoms were refined istropically.

This compound is isostructural with KCuFe(PO_4_)_2_ (Badri *et al.*, 2011) and K(Fe,Mg)(PO_4_)_2_ (Yatskin *et al.*, 2012). The structure consists of a three-dimensional assemblage of Cu_2_O_8_ units of edge-sharing CuO_5_ trigonal bipyramids, linked to each other by FeO_6_ octahedra through common corners to form crossing zigzag chains that run parallel to the [011] and [011] directions (Fig. 1). These chains are linked by the PO_4_ tetrahedra, giving rise to a three-dimensional framework.

The environments of the different coordination polyhehdra are shown in Fig. 2. Each CuO_5_ polyhedron is linked to four PO_4_ tetrahedra, by sharing three corners with three tetrahedra and one edge with the fourth, the two O atoms forming the shared edge being also shared by two FeO_6_ octahedra (Fig. 2*a*). Each FeO_6_ octahedron is corner-linked to six PO_4_ tetrahedra, two of its corners being also shared with two CuO_5_ polyhedra (Fig. 2*b*). Each P1O_4_ tetrahedron shares one edge with one CuO_5_ polyhedron and four corners, one with a CuO_5_ polyhedron and three with three FeO_6_ octahedra (Fig. 2*c*). Each P2O_4_ tetrahdron shares three corners with three FeO_6_ octahedra,the remaining corner being doubly shared with two CuO_5_ polyhedra (Fig. 2*d*). The anionic framework induced by this mode of connectivity forms quasi-elliptic tunnels along the [101] direction (Fig. 3).

From an examination of the cation–oxygen distances, the CuO_5_ polyhedron is highly deformed, with one longer Cu—O distance of 2.174 (3) Å compared to the four others [from 1.916 (3) to 2.030 (3) Å]. Such deformation has already been observed in KCuFe(PO_4_)_2_ and it can be attributed to the Jahn–Teller effect accentuated for the Cu^2+^ ions with d^9^ configuration. The FeO_6_ octahedron is also highly distorted, whith Fe—O distances varying between 1.937 (4) and 2.187 (3) Å. Corresponding mean <Fe—O> distance of 2.018 Å is close to 2.03 Å predicted by Shannon for octahedral Fe^3+^ ions (Shannon, 1976). The P—O distances within the PO_4_ tetrahedra are in the range 1.518 (3) and 1.576 (3) Å, with an overall distances of 1.540 (3) Å consistent with 1.537 Å calculated by Baur for the monophosphate groups (Baur, 1974). The Rb^+^ ions occupy a single distinct site. Their environment was determined assuming all cation–oxygen distances shorter than the shortest distance between Rb^+^ and its nearest cation. This environment (Fig. 4) is then constituted by nine O atoms, with Rb—O distances ranging from 2.887 (3) to 3.123 (3) Å.

### Experimental

Single crystals of RbCuFe(PO_4_)_2_ were grown in a flux of rubidium dimolybdate, Rb_2_Mo_2_O_7_, in an atomic ratio P:Mo = 4:1. Appropriate amounts of Rb_2_CO_3_ (Fluka, 99%), Cu(NO_3_)_2_.6H_2_O (Acros, 99%), Fe(NO_3_)_3_.9H_2_O (Fisher, 98.6%), (NH_4_)_2_HPO_4_ (Merck, 99%) and MoO_3_ (Acros, 99%) were dissolved in aqueous nitric acid and the obtained solution was evaporated to dryness. The obtained residue was homogenized by grinding and then gradually heated up to 873 K in a platinum crucible. After being reground, the mixture was melted for 1 h at 1173 K and subsequently cooled at a rate of 10 K h^-1^ down to 673 K, after which the furnace was turned off. The crystals extracted from the flux by washing with warm water, are essentially composed by dark-green plate crystals of RbCuFe(PO_4_)_2_.

### Refinement

Due to the poor quality of the crystal, anisotropic structure refinement led to non-positive definite thermal parameters for the Fe, P1, P2, O11, O13, O21 and O23 atoms. Then, all the atoms were refined istropically. The obtained largest positive and negative difference electron densities are closest to the Rb atoms.

### Crystal data

**Table:**

RbCuFe(PO_4_)_2_	*F*(000) = 744
*M**_r_* = 394.80	*D*_x_ = 3.983 Mg m^−^^3^
Monoclinic, *P*2_1_/*n*	Mo *K*α radiation, λ = 0.71073 Å
Hall symbol: -P 2yn	Cell parameters from 25 reflections
*a* = 8.054 (1) Å	θ = 2.8–26.9°
*b* = 9.906 (3) Å	µ = 13.28 mm^−^^1^
*c* = 9.140 (1) Å	*T* = 293 K
β = 115.47 (1)°	Plate, green
*V* = 658.3 (2) Å^3^	0.25 × 0.21 × 0.14 mm
*Z* = 4	

### Data collection

**Table:**

Enraf–Nonius CAD4 diffractometer	1236 reflections with *I* > \2(*I*)
Radiation source: fine-focus sealed tube	*R*_int_ = 0.046
Graphite monochromator	θ_max_ = 26.9°, θ_min_ = 2.8°
ω/2θ scans	*h* = −1→10
Absorption correction: part of the refinement model (Δ*F*) (*SHELXL97*; Sheldrick 2008)	*k* = −1→12
*T*_min_ = 0.060, *T*_max_ = 0.119	*l* = −11→10
1939 measured reflections	2 standard reflections every 120 min
1437 independent reflections	intensity decay: 1%

### Refinement

**Table:**

Refinement on *F*^2^	Primary atom site location: structure-invariant direct methods
Least-squares matrix: full	Secondary atom site location: difference Fourier map
*R*[*F*^2^ > 2σ(*F*^2^)] = 0.046	*w* = 1/[σ^2^(*F*_o_^2^) + (0.0642*P*)^2^ + 8.1747*P*] where *P* = (*F*_o_^2^ + 2*F*_c_^2^)/3
*wR*(*F*^2^) = 0.126	(Δ/σ)_max_ = 0.001
*S* = 1.08	Δρ_max_ = 3.25 e Å^−^^3^
1437 reflections	Δρ_min_ = −2.25 e Å^−^^3^
54 parameters	Extinction correction: *SHELXL97* (Sheldrick, 2008), Fc^*^=kFc[1+0.001xFc^2^λ^3^/sin(2θ)]^-1/4^
0 restraints	Extinction coefficient: 0.056 (3)

### Special details

**Table:**

Geometry. All e.s.d.'s (except the e.s.d. in the dihedral angle between two l.s. planes) are estimated using the full covariance matrix. The cell e.s.d.'s are taken into account individually in the estimation of e.s.d.'s in distances, angles and torsion angles; correlations between e.s.d.'s in cell parameters are only used when they are defined by crystal symmetry. An approximate (isotropic) treatment of cell e.s.d.'s is used for estimating e.s.d.'s involving l.s. planes.
Refinement. Refinement of *F*^2^ against ALL reflections. The weighted *R*-factor *wR* and goodness of fit *S* are based on *F*^2^, conventional *R*-factors *R* are based on *F*, with *F* set to zero for negative *F*^2^. The threshold expression of *F*^2^ > σ(*F*^2^) is used only for calculating *R*-factors(gt) *etc*. and is not relevant to the choice of reflections for refinement. *R*-factors based on *F*^2^ are statistically about twice as large as those based on *F*, and *R*-factors based on ALL data will be even larger.

### Fractional atomic coordinates and isotropic or equivalent isotropic displacement parameters (Å^2^)

**Table:**

	*x*	*y*	*z*	*U*_iso_*/*U*_eq_	
Rb	0.08146 (10)	−0.36746 (7)	0.92434 (9)	0.0187 (3)*	
Cu	0.36913 (11)	−0.11858 (8)	0.94182 (10)	0.0103 (3)*	
Fe	0.98385 (13)	0.12391 (9)	0.75520 (11)	0.0084 (3)*	
P1	1.2630 (2)	0.08995 (18)	0.1423 (2)	0.0082 (4)*	
O11	0.4087 (7)	0.0231 (5)	0.0958 (6)	0.0116 (10)*	
O12	0.2147 (7)	−0.0101 (5)	0.2460 (6)	0.0116 (10)*	
O13	0.8515 (7)	0.2895 (5)	0.7505 (6)	0.0123 (10)*	
O14	0.0942 (7)	0.1317 (5)	0.9900 (6)	0.0115 (10)*	
P2	0.1321 (2)	−0.15855 (18)	0.6387 (2)	0.0087 (4)*	
O21	0.4575 (7)	−0.2610 (5)	0.1010 (6)	0.0138 (10)*	
O22	0.1462 (7)	−0.1108 (5)	0.4869 (6)	0.0119 (10)*	
O23	0.3040 (7)	−0.2436 (5)	0.7543 (6)	0.0118 (10)*	
O24	0.1483 (7)	−0.0378 (5)	0.7545 (6)	0.0112 (10)*	

### Geometric parameters (Å, º)

**Table:**

Rb—O13^i^	2.888 (5)	Fe—O14^vii^	1.940 (5)
Rb—O21^ii^	2.947 (5)	Fe—O13	1.946 (5)
Rb—O12^iii^	2.952 (5)	Fe—O12^vi^	1.952 (5)
Rb—O21^iii^	2.969 (5)	Fe—O22^vi^	2.005 (5)
Rb—O14^iv^	3.003 (5)	Fe—O24^vii^	2.081 (5)
Rb—O12^v^	3.085 (5)	Fe—O23^viii^	2.185 (5)
Rb—O23	3.087 (5)	P1—O13^ix^	1.519 (5)
Rb—O11^iii^	3.120 (5)	P1—O14^x^	1.526 (5)
Rb—O22^v^	3.122 (5)	P1—O12^vii^	1.533 (5)
Cu—O11^ii^	1.915 (5)	P1—O11^vii^	1.557 (5)
Cu—O21^ii^	1.930 (5)	P2—O22	1.515 (5)
Cu—O23	1.993 (5)	P2—O21^iii^	1.521 (5)
Cu—O24	2.028 (5)	P2—O24	1.565 (5)
Cu—O11^vi^	2.178 (5)	P2—O23	1.576 (5)
			
O13^i^—Rb—O21^ii^	69.35 (14)	O11^ii^—Cu—O23	170.6 (2)
O13^i^—Rb—O12^iii^	112.20 (14)	O21^ii^—Cu—O23	94.0 (2)
O21^ii^—Rb—O12^iii^	176.53 (14)	O11^ii^—Cu—O24	98.0 (2)
O13^i^—Rb—O21^iii^	138.05 (14)	O21^ii^—Cu—O24	146.1 (2)
O21^ii^—Rb—O21^iii^	102.61 (12)	O23—Cu—O24	73.0 (2)
O12^iii^—Rb—O21^iii^	78.36 (14)	O11^ii^—Cu—O11^vi^	84.8 (2)
O13^i^—Rb—O14^iv^	54.85 (14)	O21^ii^—Cu—O11^vi^	112.0 (2)
O21^ii^—Rb—O14^iv^	93.87 (14)	O23—Cu—O11^vi^	93.67 (19)
O12^iii^—Rb—O14^iv^	89.52 (14)	O24—Cu—O11^vi^	100.20 (19)
O21^iii^—Rb—O14^iv^	86.08 (14)	O14^vii^—Fe—O13	88.7 (2)
O13^i^—Rb—O12^v^	48.09 (13)	O14^vii^—Fe—O12^vi^	90.8 (2)
O21^ii^—Rb—O12^v^	68.83 (13)	O13—Fe—O12^vi^	92.7 (2)
O12^iii^—Rb—O12^v^	109.70 (11)	O14^vii^—Fe—O22^vi^	176.0 (2)
O21^iii^—Rb—O12^v^	168.12 (14)	O13—Fe—O22^vi^	90.5 (2)
O14^iv^—Rb—O12^v^	102.37 (13)	O12^vi^—Fe—O22^vi^	85.3 (2)
O13^i^—Rb—O23	121.31 (14)	O14^vii^—Fe—O24^vii^	92.6 (2)
O21^ii^—Rb—O23	56.71 (14)	O13—Fe—O24^vii^	172.8 (2)
O12^iii^—Rb—O23	123.04 (13)	O12^vi^—Fe—O24^vii^	94.4 (2)
O21^iii^—Rb—O23	49.30 (14)	O22^vi^—Fe—O24^vii^	88.73 (19)
O14^iv^—Rb—O23	105.24 (13)	O14^vii^—Fe—O23^viii^	91.5 (2)
O12^v^—Rb—O23	119.56 (13)	O13—Fe—O23^viii^	85.59 (19)
O13^i^—Rb—O11^iii^	158.60 (14)	O12^vi^—Fe—O23^viii^	177.11 (19)
O21^ii^—Rb—O11^iii^	129.19 (14)	O22^vi^—Fe—O23^viii^	92.38 (19)
O12^iii^—Rb—O11^iii^	48.65 (13)	O24^vii^—Fe—O23^viii^	87.29 (19)
O21^iii^—Rb—O11^iii^	55.53 (14)	O13^ix^—P1—O14^x^	111.4 (3)
O14^iv^—Rb—O11^iii^	124.74 (13)	O13^ix^—P1—O12^vii^	106.2 (3)
O12^v^—Rb—O11^iii^	122.91 (13)	O14^x^—P1—O12^vii^	112.1 (3)
O23—Rb—O11^iii^	80.05 (13)	O13^ix^—P1—O11^vii^	108.3 (3)
O13^i^—Rb—O22^v^	98.03 (14)	O14^x^—P1—O11^vii^	110.3 (3)
O21^ii^—Rb—O22^v^	72.07 (14)	O12^vii^—P1—O11^vii^	108.4 (3)
O12^iii^—Rb—O22^v^	104.52 (14)	O22—P2—O21^iii^	112.5 (3)
O21^iii^—Rb—O22^v^	119.17 (14)	O22—P2—O24	111.3 (3)
O14^iv^—Rb—O22^v^	152.82 (13)	O21^iii^—P2—O24	110.7 (3)
O12^v^—Rb—O22^v^	51.19 (13)	O22—P2—O23	113.0 (3)
O23—Rb—O22^v^	86.70 (13)	O21^iii^—P2—O23	109.4 (3)
O11^iii^—Rb—O22^v^	80.87 (13)	O24—P2—O23	99.3 (3)
O11^ii^—Cu—O21^ii^	95.2 (2)		

Symmetry codes: (i) −*x*+1, −*y*, −*z*+2; (ii) *x*, *y*, *z*+1; (iii) *x*−1/2, −*y*−1/2, *z*+1/2; (iv) −*x*, −*y*, −*z*+2; (v) −*x*+1/2, *y*−1/2, −*z*+3/2; (vi) −*x*+1, −*y*, −*z*+1; (vii) *x*+1, *y*, *z*; (viii) −*x*+3/2, *y*+1/2, −*z*+3/2; (ix) *x*+1/2, −*y*+1/2, *z*−1/2; (x) *x*+1, *y*, *z*−1.
